# A Real-World Evidence Study Using Alberta-Population-Based Data to Describe Treatment Patterns for Metastatic Castration-Sensitive Prostate Cancer Patients (AWARENESS)

**DOI:** 10.3390/curroncol30090591

**Published:** 2023-09-01

**Authors:** Safiya Karim, Jennifer Lowther, Gabriel Gyulay, Dylan O’Sullivan, Christopher J. D. Wallis, Steven M. Yip, Darren R. Brenner, Devon J. Boyne, Winson Y. Cheung

**Affiliations:** 1 Department of Oncology, Cumming School of Medicine, University of Calgary, Calgary, AB T2N 1N4, Canadadarren.brenner@oncoutcomes.com (D.R.B.); winson.cheung@albertahealthservices.ca (W.Y.C.); 2 Medical Affairs, Bayer Inc., Mississauga, ON L4W 5R6, Canada; 3 Department of Surgical Oncology and Urology, Mount Sinai Hospital and University Health Network, University of Toronto, Toronto, ON M5S 1A1, Canada

**Keywords:** metastatic hormone-sensitive prostate cancer, treatment intensification, real-world evidence

## Abstract

Introduction: Over the past decade, the treatment of metastatic castration-sensitive prostate cancer (mCSPC) has changed significantly. Current guidelines suggest the use of androgen deprivation therapy (ADT) plus an additional systemic therapy, regardless of disease burden and risk, based on phase 1 evidence showing improved overall survival. We sought to describe treatment patterns of patients with mCSPC in the province of Alberta. Methods: This was a retrospective, population-based, cohort study of male patients aged ≥18 with mCSPC at the time of diagnosis and who initiated ADT between 1 January 2016 and 31 December 2020. Data were obtained from the Alberta Cancer Registry. Patients were assigned to an ADT-alone cohort or a treatment intensification cohort (cohorts 2–5). The primary objectives of this study were to describe baseline characteristics and the treatment of mCSPC patients who initiated ADT with or without treatment intensification. Overall survival between cohorts was a secondary objective. Descriptive statistics were used to describe differences in baseline characteristics of each cohort. Overall survival was calculated using the Kaplan–Meier method. All statistical tests were two-sided and are used to call out likely cohort differences descriptively. Results: Between 1 January 2016 and 31 December 2020, we identified a total of 960 patients with mCSPC (median age 74 years, IQR 66–82). Most patients received ADT alone (67%), followed by ADT plus abiraterone (18%), ADT plus docetaxel (12%), and ADT plus enzalutamide or apalutamide (3%). Over the study period, we observed an increase in the utilization of treatment intensification over time, in particular, the increased use of androgen-receptor-axis-targeted (ARAT) therapies. Patients who received ADT alone were older, were more likely to have more than one comorbid condition, had fewer sites of metastatic disease, and were less likely to be on opioid medications. Conclusions: In this study, we show that patients who received ADT alone as treatment for mCSPC are older, have more comorbidities, and have less extensive disease. While there has been a decline over time in the number of patients treated with ADT alone, over 50% of all patients with mCSPC continue to receive ADT alone. Further work is needed to understand barriers to treatment intensification and for knowledge translation initiatives to improve the treatment of patients with mCSPC.

## 1. Introduction

The treatment landscape of metastatic castration-sensitive prostate cancer (mCSPC) has evolved significantly over the past 10 years. Historically, patients with mCSPC were treated with androgen deprivation therapy (ADT) alone via surgical or medical castration. However, resistance to ADT develops quickly in some patients, resulting in progression to castration-resistant disease, which has an overall survival of 1–3 years [1].

Several randomized clinical trials have shown a survival benefit to adding additional systemic therapies to ADT, including docetaxel, abiraterone acetate (abiraterone), and more recently apalutamide or enzalutamide, for men with mCSPC [2,3,4,5,6]. Based on this, guidelines including those from the American Society of Clinical Oncology (ASCO) [7], European Society of Medical Oncology (ESMO) [8], as well as several national and international guidelines [9,10,11] recommend the use of ADT plus an additional systemic therapy (docetaxel, abiraterone, enzalutamide, or apalutamide) regardless of disease burden or risk. Furthermore, in patients with de novo metastatic castration-sensitive prostate cancer with high-volume disease and being offered ADT plus docetaxel, these guidelines also recommend that triplet therapy (ADT plus docetaxel plus either abiraterone and prednisone or Darolutamide) should be offered based on recent studies showing improvement in overall survival compared to ADT + docetaxel [12,13].

Recent real-world evidence studies suggest that the majority of mCSPC patients are receiving ADT alone, despite the availability of additional life-prolonging therapies [14,15,16,17]. For example, a retrospective, population-based study using the Institute for Clinical Evaluative Sciences (ICES) database of men older than 66 years with de novo mCSPC in Ontario, Canada, between 2014 and 2019 showed that approximately 78% of patients were treated with ADT alone, and this did not change significantly before and after 2017 [17]. Similar real-world studies using claims databases in the United States also show that most patients continue to receive ADT alone as initial treatment for mCSPC [14,15,16]. However, as the ICES data only include data on publicly funded drugs, there may have been under-reporting of the use additional life-prolonging agents available as part of a manufacturer-sponsored patient access program or covered by private drug insurance plans. In addition, claims databases only capture information on a subset of the entire population.

The purpose of this study was to conduct a population-based real-world analysis of treatment patterns and outcomes of patients with mCSPC receiving ADT with and without treatment intensification.

## 2. Methods

This was a retrospective, population-based cohort study that analyzed data from the Alberta Cancer Registry (ACR). The ACR includes the province’s entire population, which is estimated to be approximately 4 million people during the study period (2016–2020). All patients have access to a single-payer, universal health care system in the province. The ACR prospectively collects information on patient demographics, tumor characteristics (including cancer staging), primary treatment, and oncology facility from all individuals who resided in the province at the time of their initial, confirmed cancer diagnosis. Since cancer is a reportable disease in the province, case ascertainment is complete and accurate in the ACR within 12 months of a cancer diagnosis.

The study population consisted of male patients with prostate cancer who had metastatic disease at the time of diagnosis (i.e., de novo mCSPC) and initiated ADT between 1 January 2016 and 31 December 2020. Patients were diagnosed with de novo mCSPC up to 31 December 2019 and followed until 31 December 2020. Patients were assigned to the ADT-alone cohort (cohort 1) if they did not have the addition of the following agents within 180 days from initiation of ADT: docetaxel, abiraterone, enzalutamide, or apalutamide. Treatment intensification cohorts consisted of patients who received ADT + docetaxel (cohort 2), ADT plus abiraterone (cohort 3) or ADT plus enzalutamide (cohort 4), and ADT plus apalutamide (cohort 5). We also included a cohort that consisted of patients on triplet therapy (i.e., ADT plus either abiraterone or Darolutamide) (cohort 6), but given the dates of this study, there were no patients in this cohort. We also attempted to assess the use of radiation therapy (RT) to the prostate as per the STAMPEDE trial [18], but we were not able to differentiate the intent of the RT via administrative databases.

Use of treatment intensification was captured in the database and included all sources included publicly funded drugs and those offered through private-payer- and manufacturer-sponsored programs. Given that cases of mCSPC were only captured until the end of 2019 and the follow-up for ADT was only 180 days, only patients treated in early 2020 were captured. We therefore combined these patients treated in 2019. We analyzed treatment intensification based on the changing landscape of approved treatments during various time periods: 2016 where only docetaxel was primarily available for treatment intensification, 2017 and 2018: with the approval of abiraterone, and 2019 and beyond: with the addition of enzalutamide and apalutamide.

Baseline characteristics of the study population included age, rural residence, quartile of neighborhood annual income and education level from the 2016 census, Charleston comorbidity index, specific comorbidities, number and sites of metastatic disease at diagnosis, M staging (M1a, M1b, or M1c), and use of bone modifying and opioid medication from the Pharmaceutical Information Network (PIN) database.

The primary objective of this study was to describe baseline characteristics and treatment of mCSPC patients who initiated ADT with or without treatment intensification. The secondary objectives were to describe overall survival in the ADT-alone cohort and the treatment intensification cohorts. An exploratory objective was to describe treatment patterns beyond initial therapy for mCSPC.

Descriptive statistics were summarized for baseline characteristics for each cohort. To compare the distribution of baseline characteristics between cohorts, *p*-values corresponding to *t*-tests for continuous variables and chi-square tests for categorical variables are presented, as are standardized mean differences in which values > 0.1 are indicative of an imbalance [19]. A Sankey diagram was generated to provide an overview of treatment patterns and treatment sequences among the different cohorts. Overall survival was calculated using the Kaplan–Meier method. Overall survival was defined as the time from the start of ADT for the ADT-alone cohort, and from the start of treatment intensification for those with treatment intensification, to account for immortal time bias, until death from any cause. All cell counts fewer than 10 were suppressed (reported as <10) due to data privacy regulations. All statistical analyses were conducted using the R computing framework [20]. All statistical tests were 2-sided and are used to call out likely cohort differences descriptively.

This study was designed, analyzed, and reported in accordance with the STROBE (Strengthening the Reporting of Observational Studies in Epidemiology) statement [21]. This study was approved by the Health Research Ethics Board of Alberta Cancer Committee (HREBA.CC-22-0013).

## 3. Results

**Figure 1:**
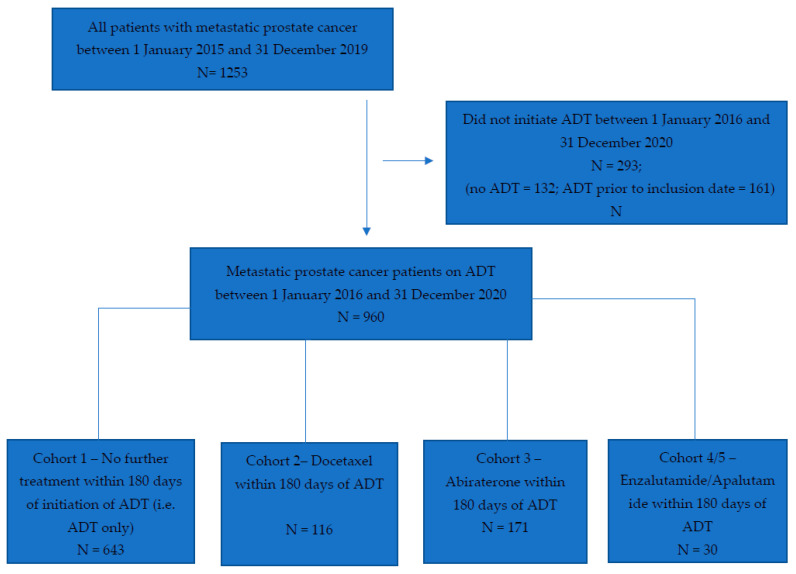
Identification of patients with de novo metastatic castration-sensitive prostate cancer.

Between 1 January 2016 and 31 December 2020, we identified a total of 960 patients with mCSPC (Figure 1). Baseline characteristics of the entire population and by cohort are shown in Table 1. The median age of the entire cohort was 74.0 years (IQR 66.0–82.0). Most patients received ADT alone (67%, 643/960), followed by ADT plus abiraterone (18%, 171/960), ADT plus docetaxel (12%, 116/960) and ADT plus enzalutamide or apalutamide (3%, 30/960). The number of patients initiating apalutamide was too few to allow reporting as a separate cohort; therefore, cohorts 4 (ADT + enzalutamide) and 5 (ADT + apalutamide) were combined.

Patients who received ADT alone were older (*p* < 0.001; SMD = 0.812); were more likely to have more than one comorbid condition (*p* < 0.001; SMD = 0.329), including diabetes (*p* = 0.010; SMD = 0.189) and cardiovascular disease (*p* < 0.001; SMD = 0.346); had fewer sites of metastatic disease at diagnosis (*p* < 0.001; SMD = 0.455); and were less likely to be on opioid medications (*p* = 0.011; SMD = 0.178). Compared to patients on ADT plus docetaxel, patients on ADT plus abiraterone, enzalutamide, or apalutamide (cohorts 3–5) were older in age (*p* < 0.001; SMD = 0.531), more likely to have one or more comorbid conditions (*p* = 0.005; SMD = 0.358), more likely to receive radiation (*p* = 0.046; SMD = 0.256), and more likely to be diagnosed with mCSPC in more recent years (*p* < 0.001; SMD = 1.390). These characteristics remained significantly different when restricting the analysis to 2017 and later (i.e., in years where there was an increased proportion of patients in cohorts 3–5).

### 3.1. Changes in Treatment Pattern over Time

Table 2 shows the changes in the percentage of patients within each treatment cohort between 2016 and 2019, divided into relevant treatment eras. Over time, the percentage of patients who received ADT alone decreased (77% in 2016 to 53% in 2019, *p* < 0.001). There was a slight decrease in patients who received docetaxel between 2016 and 2017/2018 (17.5% vs. 13.3%), but the largest decline occurred between 2017/2018 and 2019 (13.3% to 4.4%). This parallels a similar rate of increase in the use of abiraterone, from 5% in 2016 to 17% in 2017/2018 and to 33% in 2019. The use of enzalutamide or apalutamide in combination with ADT was negligible prior to 2019 due to the lack of access at the time.

### 3.2. Sequencing of Therapies

**Figure 2:**
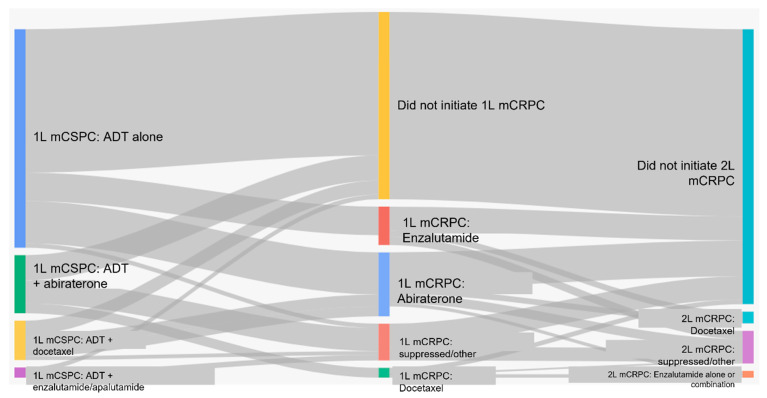
Sequencing of therapies from mCSPC to mCRPC.

Figure 2 shows the sequencing of therapies from mCSPC to metastatic castration-resistant prostate cancer (mCRPC) for each cohort. In patients who received ADT alone for mCSPC, 66% (422/643) did not receive any further therapy. Thirty-four percent (221/643) of patients received first-line therapy for mCRPC, most commonly abiraterone (56%) or enzalutamide (38.0%); none of these patients received docetaxel. Ten percent (66/643) of the entire cohort initiated second-line treatment and 4% (24/643) received third-line treatment. There were no patients who received four lines of therapy for mCRPC (Table 3).

In those mCSPC patients who received ADT plus additional life-prolonging therapies (cohorts 2–5), 58% (183/317) did not receive any further therapy. Forty-two percent (134/317) of patients received first-line therapy for mCRPC and 20% of all patients (64/317) initiated second-line therapy. Five percent (16/317) received third-line therapy. There were no patients who received four lines of therapy for mCRPC (Table 4).

### 3.3. Overall Survival

Median follow-up times were similar in patients who received ADT alone (22.32 months (IQR: 12.15–35.87)) and those who received treatment intensification (24.43 months (IQR: 17.52–33.04)). The median overall survival (mOS) of all patients with de novo mCSPC was 28.7 months (95% CI 26.5–31.2). In those treated with ADT alone, mOS was 27.3 months (95% CI 24.3–29.8), and in those treated with ADT plus other life-prolonging therapies, mOS was 31.3 months (95% CI 28.5–37.7) (Supplementary Figure S1).

## 4. Discussion

In this study, we assessed the treatment patterns and outcomes of males with mCSPC in Alberta who received ADT with and without treatment intensification. There are several important findings. First, and most importantly, we observed an increase in the utilization of treatment intensification over time. In particular, there was an increased use of androgen-receptor-axis-targeted (ARAT)-based treatment intensification. Second, across the whole study period, patients who received ADT alone were older, were more likely to have more than one comorbid condition, including diabetes and heart disease, and had less extensive metastatic disease. Third, patients who received ADT alone were less likely to initiate subsequent lines of therapy and never receive docetaxel as part of their treatment.

Our findings are consistent with other real-world data studies of the treatment of mCSPC. In a similar health system in Ontario, Canada, men who were treated with ADT alone were, on average, 1.6 years older than men receiving ADT plus abiraterone and 5.74 years older than those receiving ADT + docetaxel [17]. In addition, the median PSA of patients on ADT alone was significantly lower than those treated with ADT plus docetaxel or ADT + abiraterone, which is likely a surrogate for less extensive metastatic disease. Similarly, Heath et al. showed that there was a steady increase in the administration of ADT alone with age in a large US-based Medicare database (40% age ≤ 59 vs. 55% age ≥ 80) [15]. Conversely, in a large database study of the Veterans Health Administration in the US, men who received ADT alone were slightly younger than those who received ADT plus abiraterone but also had less disease burden in terms of baseline PSA and number of sites of metastatic disease [16].

Other studies have observed similar changes in treatment patterns over time with the decline in use of ADT alone and an increase in treatment intensification. Freedland et al. showed that between 2014 and 2017, patients in the US Veterans Health Administration database on ADT alone decreased from 66 to 60% while those on ADT + docetaxel and ADT + abiraterone increased (3% to 9% and 1% to 15%, respectively) [16].

While our study showed a decline in the use of ADT + docetaxel over time, this may have been due to the differences in the years of the study period as well as by the mix of patients on the different studies (i.e., high volume vs. low volume). Our study showed that in 2017/2018, there was a similar proportion of patients who received treatment intensification with docetaxel and abiraterone (13.3% and 16.7%, respectively). The largest drop in the use of docetaxel as intensification was between 2017/2018 and 2019, when several other ARAT agents were available. The study by Ryan et al. also showed that the use of docetaxel decreased over similar time periods (from 7% in 2015–2017 to 4% in 2018–2019).

A unique aspect of our study is that we were able to determine the subsequent treatments in mCSPC patients who received ADT alone and those who received treatment intensification. In mCSPC patients on ADT alone compared to mCSPC patients who received treatment intensification, a significantly smaller proportion of patients initiated first-line therapy for metastatic castration-resistant prostate cancer (mCRPC) (34% vs. 42%) and second-line (10% vs. 20%) therapy. While no patient who received ADT alone in the mCSPC setting received subsequent treatment with docetaxel when they became castration-resistant, 32% of patients did receive first-line treatment in the mCRPC setting with either enzalutamide or abiraterone. This suggests that patients may have been fit enough to receive these treatments in the mCSPC setting but did not receive them up front at the time of ADT initiation.

While many real-world studies show a decline in use of ADT alone over the past ten years, upward of 40% of patients with mCSPC continue to receive ADT alone as upfront treatment [12,13,14,15]. Several reasons for this have been proposed including a lack of awareness amongst physicians, communication barriers between physicians and patients, a lack of drug access, medical comorbidities, racial or social disparities, and patient choice, to name a few [15,22]. In addition, Heath et al. showed that treatment intensification varied by provider [15]. In a large longitudinal database of prostate cancer patients in the United States, ADT monotherapy was prescribed more frequently by urologists than oncologists between 2015 and 2021 (69% vs. 43% on average, respectively). Similarly, ADT monotherapy was used more frequently at a large urology group practice compared to a large national cancer institute in the same region (79% vs. 51% on average, respectively) [15]. This signifies that educational initiatives may need to be designed to target urology providers who manage patients with mCSPC.

Our study should be interpreted in the context of certain limitations. First, the use of administrative datasets can be prone to error in misclassification, and it is possible that some patients with mCSPC were miscoded. In addition, we were unable to assess whether patients had high- vs. low-volume disease or high- vs. low-risk disease. However, we were able to assess the sites of metastatic disease and the M stage, which may be a surrogate for volume of disease. Furthermore, we were unable to capture a patient’s performance status and therefore unable to determine whether a patient was suitable for treatment intensification (i.e., ECOG <=2). Finally, patients who received ADT alone may not have progressed on to mCRPC, due to deaths (59.4% for ADT vs. 44.8% for other cohorts) and censoring, explaining the higher proportion of patients who did not receive subsequent therapies. However, the median follow-up times were similar between the ADT-alone cohort and those who received treatment intensification. These limitations should be weighed against the strengths of this study, which include a large sample size and inclusion of all patients with mCSPC in the province, including those who received treatment via a patient access program.

## 5. Conclusions

The field of oncology strives to be evidence-based and to apply the results of clinical trials into practice. However, as this study and several others describing treatment patterns of men with mCSPC have shown, the uptake of novel life-prolonging therapies in the real world is lagging despite level 1 evidence showing improved overall survival. While we may not be able to predict the optimal percentage of patients who should receive treatment intensification, it is reasonable to assume it should be higher than the reported rate of 47% from the most recent year included in this study. In addition, with recent studies showing improved overall survival in patients with mCSPC with triplet therapy (i.e., ADT plus docetaxel plus abiraterone or darolutamide) [11,12], it is important that patients are offered optimal treatment intensification up front, if appropriate. If the improvements in survival shown in clinical trials of patients with mCSPC are going to be achieved in the real world, further work must be performed to understand the reasons that patients do not receive treatment intensification and to address these barriers through multi-pronged, provider-specific knowledge translation initiatives.

## Figures and Tables

**Table 1:** Baseline characteristics of 960 patients with mCSPC stratified by ADT cohort.

Variable	Cohort 1 ADT Alone	Cohort 2 ADT + Docetaxel	Cohort 3 ADT + Abiraterone	Cohort 4/5 ADT + Enzalutamide or Apalutamide	*p*-Value *	SMD *
	(n = 643)	(n = 116)	(n = 171)	(n = 30)		
Demographics						
Age, years (mean (SD))	75.91 (10.26)	65.26 (8.01)	70.03 (8.95)	68.30 (9.01)	<0.001	0.611
Socioeconomic Status						
Rural Residence (%)	196 (30.5)	26 (22.4)	49 (28.7)	<10	0.367	0.097
Categories Neighborhood Annual Household Income (%)					0.31	0.283
Q1 (lowest income level: <USD 36,000)	162 (25.2)	29 (25.0)	44 (25.7)	<10		
Q2 (USD 36,000 to <USD 43,500)	164 (25.5)	27 (23.3)	43 (25.1)	<10		
Q3 (USD 43,500 to USD 53,700)	159 (24.8)	21 (18.1)	48 (28.1)	11 (36.7)		
Q4 (highest income level: >USD 53,700)	157 (24.5)	39 (33.6)	36 (21.1)	<10		
Categories of Proportion of Neighborhood Resident who achieved a high school education or greater (%)					0.312	0.255
Q1 (lowest education level: <0.73)	169 (26.3)	25 (21.6)	43 (25.1)	<10		
Q2 (0.73 to <0.81)	169 (26.3)	20 (17.2)	43 (25.1)	<10		
Q3 (0.81 to 0.88)	150 (23.4)	36 (31.0)	44 (25.7)	<10		
Q4 (highest education level: >0.88)	154 (24.0)	35 (30.2)	41 (24.0)	<10		
Comorbidity						
Charlson Comorbidity Index (%)					<0.001	0.299
1+	258 (40.1)	18 (15.5)	53 (31.0)	<10		
Diabetes (%)	128 (19.9)	10 (8.6)	26 (15.2)	<10	0.023	0.170
Cardiovascular Disease (%)	89 (13.8)	<10	10 (5.8)	<10	<0.001	0.260
Metastatic Sites						
Number of Metastatic Sites at Diagnosis (%)					<0.001	0.272
1	533 (79.9)	70 (60.3)	100 (57.5)	21 (67.7)		
2+	132 (20.5)	46 (39.7)	74 (43.3)	10 (33.3)		
Sites of Metastasis at Diagnosis						
Osseous (%)	558 (86.8)	106 (91.4)	156 (91.2)	29 (96.7)	0.112	0.186
Lymph nodes (%)	169 (26.3)	42 (36.2)	79 (46.2)	<10	<0.001	0.245
Pulmonary (%)	32 (5.0)	13 (11.2)	12 (7.0)	<10	0.028	0.279
M Stage					0.09	0.297
M1a	70 (10.9)	<10	12 (7.0)	<10		
M1b	510 (79.3)	90 (77.6)	135 (78.9)	27 (90.0)		
M1c	63 (9.8)	>16 (>13.8)	24 (14.0)	<10		
Medications						
Bone health agents	91 (14.2)	28 (24.1)	24 (14.0)	<10	0.047	0.14
Opioids	163 (25.3)	35 (30.2)	61 (35.7)	10 (33.3)	0.048	0.124

* SMD: Standardized Mean Difference. SMD and *p*-values are provided to help call out likely cohort differences descriptively. Note: if cell <10, percentage was estimated removing those patients from cohorts 4/5.

**Table 2:** Changes in the percentage of patients within each treatment cohort by year (2016 vs. 2017/2018 vs. 2019 *).

Cohorts	Year		
	2016	2017/2018	2019 *
Total	234	478	240
Cohort 1 ADT alone	182 (77.8%)	334 (69.8%)	127 (52.9%)
Cohort 2	41 (17.5%)	64 (13.3%)	11 (4.6%)
ADT + Doectaxel			
Cohort 3	11 (4.7%)	80 (16.7%)	80 (33.3%)
ADT + Abiraterone			
Cohort 4/5	<10	<10	22 (9.2%)
ADT + Enzalutamide or Apalutamide			

Note: if cell <10, percentage was estimated by removing those patients from cohorts 4/5. * Includes some patients that received initial treatment in 2020.

**Table 3:** Attrition rate for each line of therapy for cohort 1 (ADT alone).

	Number of Patients Still Alive	Number of Patients Initiating a New Line of Therapy	%
Start of mCSPC	643	221	34.4
Start of 1st line CRPC	221	66	29.9
Start of 2nd line CRPC	66	24	36.4
Start of 3rd line CRPC	24	0	0.0

**Table 4:** Attrition rate for each line of therapy for cohorts 2–5.

	Number of Patients Still Alive	Number of Patients Initiating a New Line of Therapy	%
Start of mCSPC	317	134	42.3
Start of 1st line CRPC	134	64	47.8
Start of 2nd line CRPC	64	16	25.0
Start of 3rd line CRPC	16	0	0.0

## Data Availability

Data are available from the corresponding author upon reasonable request.

## Supplementary Materials

The following supporting information can be downloaded at: https://www.mdpi.com/article/10.3390/curroncol30090591/s1, Figure S1: Kaplan Meier Curve for Overall Survival for all patients, patients in cohort 1 (ADT alone), and patients in cohorts 2–5 (ADT plus additional life prolonging therapies).
