# Supplementary material for: A Real-World Evidence Study Using Alberta-Population-Based Data to Describe Treatment Patterns for Metastatic Castration-Sensitive Prostate Cancer Patients (AWARENESS)
Source: Curr Oncol. 2023 Sep 1;30(9):8149–58. doi: 10.3390/curroncol30090591 (PMC10528640; doi:10.3390/curroncol30090591)

Supplemental Figure S1. Kaplan Meier Curve for Overall Survival for all patients, patients in cohort 1 (ADT alone), and patients in cohorts 2-5 (ADT plus additional life prolonging therapies)

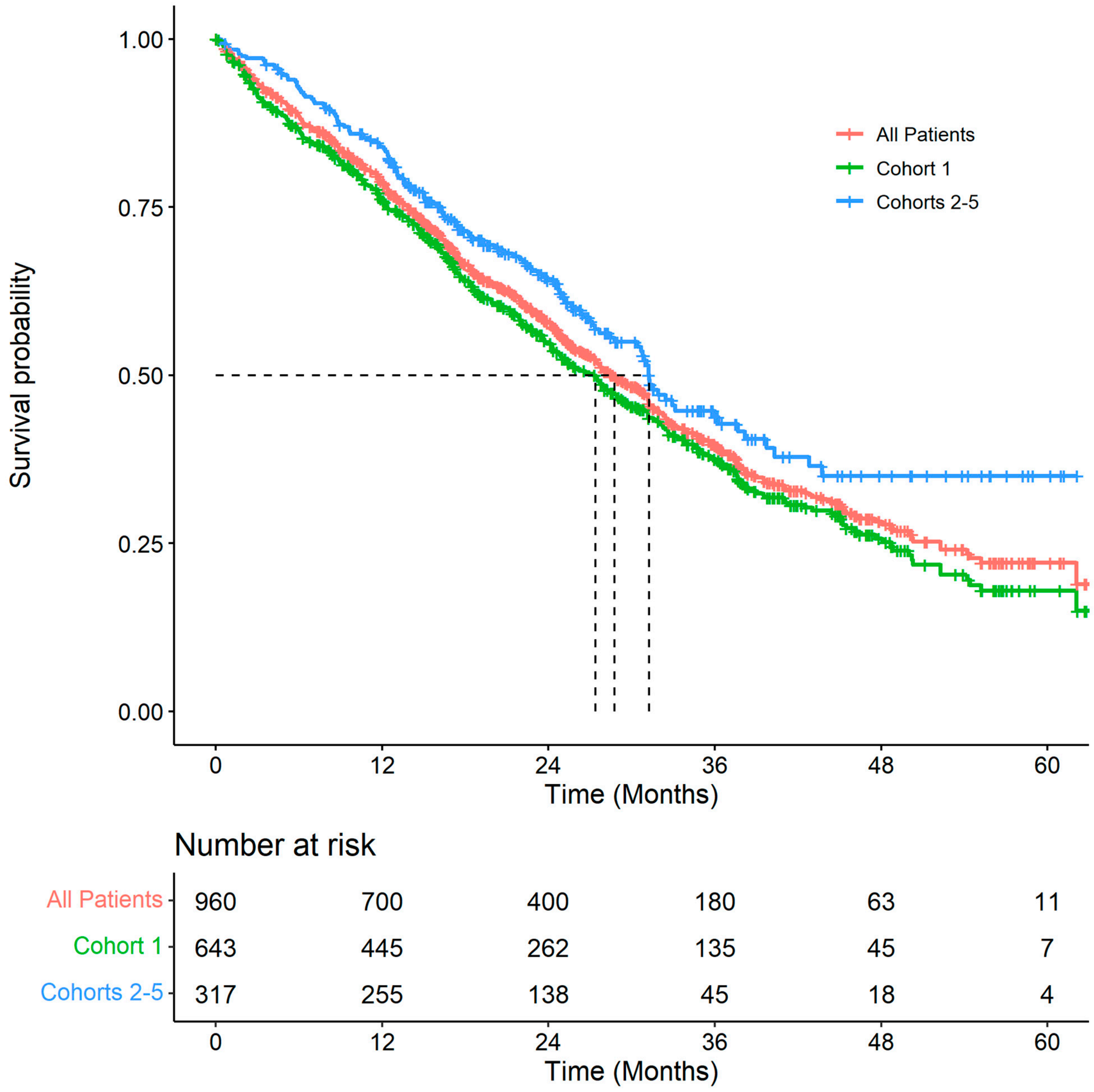

Supplement: Supplementary file 1 [file curroncol-30-00591-s001.zip › curroncol-2564541-supplementary.pdf]
